# The crucial role the field epidemiology training program played in preparedness and response to the COVID-19 pandemic in Sierra Leone, January 2020 to August 2022

**DOI:** 10.3389/fpubh.2025.1566824

**Published:** 2025-05-09

**Authors:** Gebrekrstos Negash Gebru, Alden Keith Henderson, Adel Hussein Elduma, James Sylvester Squire, Mohamed Alex Vandi, Daphane Moffett, Monique Foster

**Affiliations:** ^1^African Field Epidemiology Network, Kampala, Uganda; ^2^Department of Public Health Sciences, University of Hawaii, Kaneohe, HI, United States; ^3^National Public Health Agency, University of Sierra Leone, Freetown, Sierra Leone; ^4^Centers for Disease Control and Prevention, Atlanta, Georgia

**Keywords:** field epidemiology, COVID-19, Sierra Leone, preparedness, response, work force development

## Abstract

**Background:**

On January 30, 2020, the World Health Organization declared COVID-19 a Public Health Emergency of International Concern (PHIEC). On March 11, 2020, it was characterized as a pandemic, prompting the Government of Sierra Leone to implement response plans. The first case in the country was reported on March 31, 2020. To build resilient public health systems after the Ebola crisis, the Sierra Leone Field Epidemiology Training Program (SLFETP) was launched in 2016 with funding from the U.S. CDC in collaboration with the Ministry of Health and the African Field Epidemiology Network (AFENET). The program started at the FETP Frontline level, a 3-month in-service training program, followed by the FETP Intermediate, a 9-month in-service training program launched in 2017. Both levels adopted the CDC curriculum to the local context. The curriculum consists of classroom modules focusing on surveillance, outbreak investigation, and field projects. The SLFETP graduates and trainees were deployed to assist in COVID-19 response efforts. While reports indicate the SLFETP's contributions to COVID-19 preparedness and response, the specific roles of its graduates and trainees remain undocumented. This paper outlines their crucial involvement during the pandemic in Sierra Leone.

**Methods:**

We reviewed 12 documents from the SLFETP, including work plans, outbreak investigation reports, and success stories, to assess the FETP's contributions during the COVID-19 pandemic. We interviewed graduates and trainees about their roles and conducted discussions with stakeholders and FETP staff to explore the FETP's role during the pandemic's preparedness and response phases. A thematic analysis was performed.

**Results:**

The SLFETP played a critical role during the preparedness and response phase of the COVID-19 pandemic. The trainees and graduates enhanced the surveillance system and led key response pillars, such as coordination, surveillance, and quarantine. SLFETP supported districts by building their capacity, especially in the district surveillance pillar, to conduct case investigations, contact tracing, quarantine monitoring, and data management.

**Conclusions:**

The graduates and trainees reportedly played critical roles in key response pillars across the country in the preparedness and response phase of the COVID-19 pandemic. These gains should be maintained and scaled up to build a strong and resilient public health workforce in Sierra Leone, which is crucial for preparedness and response to future outbreaks.

## Background

On January 30, 2020, the World Health Organization (WHO) declared the coronavirus disease 2019 (COVID-19) a Public Health Emergency of International Concern (PHIEC). On March 11, 2020, it was declared as a global pandemic (1, 2). On March 31, 2020, the first case of COVID-19 was reported in Freetown, the capital of Sierra Leone. As of August 4, 2022, there were 7,736 COVID-19 confirmed cases of COVID-19 and 125 deaths reported in Sierra Leone (3, 4). Having experienced the deadliest Ebola outbreak on record in 2014–2015, which resulted in 11,310 deaths across Guinea, Liberia, and Sierra Leone due to delayed outbreak detection and a shortage of skilled field epidemiologists, there were concerns Sierra Leone would face similar challenges during the COVID-19 pandemic (5, 6).

Field Epidemiology Training Programs (FETPs) contributed to many countries improving their epidemiologic capacity to identify, investigate, respond, and communicate about public health threats including COVID-19 (7–12). The Ebola outbreak in 2014–2015 demonstrated that Sierra Leone needed trained field epidemiologists. Such field epidemiologists could support data collection and analysis, interpretation, and communication to increase the timely collection and use of public health data for decision-making, improve the quality and use of surveillance data at the national and district levels of the health system, and strengthen the capacity to respond to outbreaks and other public health threats (6).

As a part of the post-Ebola recovery efforts to build a strong and resilient public health system, the U.S. Centers for Diseases Control and Prevention (CDC) through implementing partners, the African Field Epidemiology Network (AFENET), and eHealth Africa, supported the Sierra Leone Ministry of Health (MoH) to establish a Frontline level FETP in 2016 followed by an Intermediate level in 2017 (6). The program was housed under the Directorate of Health Security and Emergencies (DHSE), Ministry of Health (MoH) which has now transitioned to become the National Public Health Agency (NPHA).

During the inception of the program, AFENET was the technical implementing partner, which included hiring mentors, developing a training plan, course implementation, and supervising the overall quality of the program. eHealth was responsible for administrative activities such as organizing workshops and procurement of training materials. The program aims to fill and strengthen Sierra Leone's public health workforce's capacity to apply epidemiological skills to reduce the threat of infectious diseases and timely respond to public health emergencies.

There are three tiers for FETP: a 3-month in-service training (FETP-Frontline), which targets public health officers at the lower level of the healthcare system; a 9 to 10-month in-service training that focuses on strengthening epidemiologic capacity at the middle level of the healthcare system, such as provinces, states, and governorates (FETP-Intermediate), and a 2-year full-time training that targets the national and middle level of the healthcare system (FETP-Advanced) (13).

The SLFETP implements the FETP-Frontline and the FETP-Intermediate. These programs recruit government-employed public health professionals from the MoH, Ministry of Agriculture (MoA), Ministry of Defense (MoD), and Environmental Protection Agency (EPA) to promote a multidisciplinary One Health approach. Eligibility for FETP-Frontline requires experience in disease surveillance and a supervisor's recommendation. For FETP-Intermediate, applicants must have a BSc in public health or a related field, 2 years of relevant work experience, be no older than 54, and commit to serving 2 years in government after graduation. The FETP faculty reviews applications based on eligibility criteria and recommendations. Shortlisted candidates are invited for written and oral interviews; their scores determine final selection. The program regularly assesses trainees' performance using a standard checklist for each competency, which includes evaluation of surveillance systems, investigation and response to disease outbreaks, data analysis, epidemiologic studies or surveys, and preparation of abstracts for scientific conferences, as well as oral or poster presentations.

As of October 2024, SLFETP graduated 317 public health professionals in FETP-Frontline and 103 in FETP-Intermediate. The FETP-Intermediate level has received accreditation from the Training Programs in Epidemiology and Public Health Interventions Network (TEPHINET) for its excellence in field epidemiology training, making it one of the first FETP-Intermediate programs to achieve global recognition (14). While FETP programmatic reports showed the contribution of the SLFETP in preparedness and response to the COVID-19 pandemic, the role and extent of the SLFETP trainees and graduates and their contributions to the response have not been formally described.

This paper describes the role, contributions, and involvement of the Sierra Leone FETP graduates, trainees, and technical staff during the preparedness and response phase of the COVID-19 pandemic. This will help the government and implementing partners understand the value of present and future investment in FETP in Sierra Leone and other similar settings in Africa.

## Methods

We employed a qualitative exploratory, descriptive, contextual study design to describe the roles and responsibilities of the FETP's trainees, graduates, and staff during the pandemic's preparedness and response phases.

From August 1 to December 15, 2022, we conducted desk reviews of data available to the SLFETP, including three work plans for training and activities, five outbreak investigation technical reports, and four success stories to document and describe the contributions of the FETP during the COVID-19 pandemic preparedness (January to March 2020) and response (March 2020 to August 2022) phases. At the time of this review, the above documents were the only available documents for review, and no other additional documents were available. In addition to the desk review, we also conducted interviews with FETP trainees, graduates, technical staff, and key stakeholders, as highlighted below.

### Sample size, and sampling techniques

The sample size for this study was determined based on the concept of category saturation (15). This principle suggests that data collection is adequate when new information stops emerging.

We also considered the scholarly argument that qualitative research can achieve saturation with relatively small sample sizes, typically ranging from 9 to 17 interviews or four to eight focus group discussions (15).

Considering these factors, we established the sample size for interviews based on category saturation. We randomly recruited 24 FETP trainees and graduates: 12 graduates from the Frontline Epidemiology Training Program (FETP), 10 graduates and trainees from the Intermediate FETP—also, four FETP technical staff members, and six key stakeholders. The key stakeholders served as program managers, directors, and technical coordinators across various response pillars during the pandemic. The purpose of this recruitment was to assess the role of the FETP during the preparedness and response phases of the pandemic, and the stakeholders were selected purposefully based on their roles and responsibilities during the pandemic.

#### Interviews and focus group discussions

We conducted key informant interviews with program managers, directors, outbreak supervisors, and four sessions of focus group discussions, each consisting of six to eight participants with FETP trainees, graduates, and technical staff—to explore the contributions of FETP trainees and graduates during the COVID-19 pandemic's preparedness and response phases. Sampling and data collection continued until redundancy in information was reached for each theme, allowing us to gain deeper insights regarding the FETP's role across different response pillars.

#### Data analysis

We used Epi-info version 7.2 to calculate frequencies and proportions for quantitative data. We used NVivo version 15 to summarize and perform thematic analyses of qualitative data. We also described the role of SLFETP trainees and graduates in responding to outbreaks or public health emergencies due to pathogens other than COVID-19, which occurred during the COVID-19 pandemic.

## Results

A desk review of documents, along with key informant interviews with and focus group discussions with FETP trainees, graduates, and staff, revealed that the FETP graduates, and technical staff were actively involved in developing multiple preparedness and response plans prior to the importation of COVID-19 into Sierra Leone. They also played a crucial role in implementing these plans when COVID-19 was first identified, and community transmission began in the country. The following sections detail the roles and responsibilities of the Sierra Leone FETP graduates, trainees, and technical staff in relation to pre-COVID-19 detection and transmission, as well as their actions during the intra-COVID-19 phase.

### Role of SLFETP trainees, graduates, and faculty staff during the preparedness phase of the COVID-19 pandemic (before the first case in Sierra Leone)

#### Coordination of the preparedness activities

The National Public Health Emergency Operation Center operated at level 2 of 4 during the preparedness phase of the COVID-19 pandemic in Sierra Leone (3). Level 2 is a response phase during which response measures involve many sectors and pillars. During this phase, measures include conducting a readiness assessment using the WHO guidelines (16), strengthening preparedness and response capacity through training, and setting up quarantine facilities for travelers. The Government of Sierra Leone (GoSL) implemented these activities through the National COVID-19 Emergency Response Committee (NACOVERC) (3).

The SLFETP started its preparedness activities before COVID-19 cases occurred in Sierra Leone. FETP faculty and mentors were members of the national preparedness task force and supported coordination, surveillance, points of entry, and laboratory pillars. SLFETP-Intermediate graduates were appointed by the District Medical Officers as the focal points for COVID-19 surveillance and response activities at the district level. The SLFETP Program Director and Resident Advisor liaised with the NACOVERC leadership in terms of policy matters. SLFETP Intermediate graduates led the disease surveillance and response departments at the District Health Management Teams.

#### Key activities

The SLFETP faculty developed protocols for quarantine, case investigation, and contact tracing, in addition to guidelines and tools for preparedness efforts. They also developed contact follow-up forms, passenger locator forms, screening algorithms for the PoE, a national quarantine database, and monitoring forms. SLFETP faculty developed training materials for case investigation, contact tracing, quarantine, and PoE. The FETP faculty reoriented 25 FETP intermediate graduates to support preparedness activities, specifically coordination, surveillance, laboratory, infection prevention and control, and other response pillars. The materials were also used to train more than 300 public health professionals and healthcare workers primarily involved in surveillance, data management, risk communication and social mobilization, and public health laboratory—the training aimed to prepare them to respond to the pandemic.

Fourteen FETP-Intermediate graduates participated in the COVID-19 risk assessment and the development of preparedness and response plans. They also participated in the development of the COVID-19 surveillance strategic plan. They provided technical assistance for adapting to the local context of the COVID-19 case definitions, case notification forms, contact tracing standard operating procedures, and other forms. They supported the development of a flow chart to identify contacts and suspected cases at the point of entry. Some of the documents developed during the response phase were:

1. A plan for decentralizing the national COVID-19 response to district levels.
2. Standard Operating Procedures for unique ID generation for cases.
3. An organogram and Terms of Reference for response pillars.
4. Guidelines for safe meetings and events during the pandemic.
5. A preparedness plan for lockdown surveillance.
6. An enhanced surveillance strategy and implementation plan.
7. A checklist for evaluating quarantine facilities.
8. A risk communication strategy.
9. A reporting tool for quarantine activities.

The SLFETP faculty and graduates developed and distributed a standard case definition for COVID-19 in accordance with the WHO interim case definition, the activation of different acute respiratory infection surveillance systems, the development and updating the standard reporting forms for COVID-19 cases and their contacts, the development of a national plan for prevention of spread and control of the COVID-19 pandemic, and reviewed the national capacity for confronting COVID-19 pandemic using WHO assessment tools.

### Point of entry screening

After the COVID-19 pandemic was declared a Public Health Emergency of International Concern by the WHO, the GoSL immediately started implementing preparedness activities at three main point of entry to prevent the importation of COVID-19 cases into the country. SLFETP trainees and graduates joined the MoH and other partners to conduct COVID-19 capacity assessments of point of entry at Lungi International Airport and two ground crossing points at the borders with Liberia (Gendema) and Guinea (Gbalamuya). The SLFETP graduates developed protocols for passenger quarantine, a locator/screening form, monitoring tools, a COVID-19 screening algorithm, and a reporting tool for quarantine activities. SLFETP graduates and trainees of the FETP Intermediate, with technical support of mentors, designed passengers' screening flow to allow social distancing at arrival and a referral system of suspected COVID-19 cases from PoE to the nearest health facility.

In the pre-epidemic preparedness phase, SLFETP deployed 17 graduates to screen and monitor quarantined passengers from highly affected countries to prevent the importation of COVID-19 cases in the country. The government instituted a 14-day quarantine on all travelers from China at the beginning of the preparedness phase and later included travelers from other countries in Asia and Europe before the borders closed on March 27, 2020. All borders, including land crossing and air, were closed for 4 months before opening on July 22, 2020. SLFETP graduates were trained and deployed at the various PoE to screen incoming travelers and monitor them for 14 days. For travelers with suspected COVID-19, SLFETP graduates followed up with interviews, sample collection, health education, isolation, identifying contacts, and linking COVID-19 confirmed cases to health facilities.

“*We were constrained with shortage of staff at several POEs to screen passengers, but now we have 56 Port Health Assistants including volunteers from the Seaport who are trained/oriented by FETP on passenger screening, data capture, referral and reporting skills and we have deployed them within South-East boarder in Kenema, Kailahun, Kono and Pujehun districts. After the orientation, they showed massive improvement in reporting and data collection,”* A senior surveillance officer of POEs indicated in his statement during a cross-border surveillance meeting at the Lungi Airport.

#### Surveillance

SLFETP graduates developed and implemented an event- and case-based COVID-19 surveillance mechanism at the national and subnational disease surveillance and response units where contact tracing and screening of suspected cases were conducted.

### Role of SLFETP trainees, graduates, and faculty during the response phase of the pandemic (after the first case in Sierra Leone)

When the first COVID-19 case was identified in Sierra Leone on March 31, 2020 (3), the country moved to the response phase. At the national level, the Emergency Operation Center was activated to level 3 of 4. At this level, a national incident manager was appointed, resources were mobilized, and Ministries, Departments, and Agencies (MDAs) were involved in coordinating local, national, and international multi-agency support through key pillars such as technical coordination, surveillance, laboratory, infection prevention and control, case management, communication, psychosocial support, and logistics.

The MOH requested that the SLFETP deploy its trainees, graduates, and faculty to support the response. Consequently, all core SLFETP training activities were suspended immediately, and all 15 trainees and 40 graduates of the SLFETP-Intermediate were deployed to support the COVID-19 response. SLFETP was most involved with the technical coordination pillar, including the surveillance and sub-pillars. These pillars included case investigation, contact tracing, quarantine, and data management. Ten FETP graduates, and trainees were deployed to join the national-level technical coordination pillar. SLFETP trainees and graduates were deployed to support COVID-19 response activities at various levels of the healthcare system: national, district, and sub-district levels.

The SLFETP faculty were deployed to provide technical support to the sub-pillars of the national response task force. The FETP faculty, including the resident advisor and international mentors, were assigned as technical advisors to each sub-pillar of the surveillance and the national technical coordination pillar. They helped develop SOPs and guidelines on case investigation, contact tracing, quarantine and isolation, surge surveillance, setting up response structure, rumors, schools, prisons, lockdown, and containment strategy. The FETP program director said on the role of FETP “*as the world is still challenged with newly emerging and re-emerging infectious diseases such as the recent Coronavirus (COVID-19), nowadays, the importance of FETP is more evident than ever*.”

### Strengthening points of entry

The SLFETP trainees provided 24/7 support to the response activities in all surveillance sub-pillars at points of entry. This involved screening passengers, quarantining and testing travelers from high-risk countries, isolating suspected cases, and following up with contacts.

The SLFETP trainees were also involved in case follow-up, data management, and developing tools for surveillance at points of entry, such as questionnaires for case detection. SLFETP trainees assessed the preparedness and response at major PoE, handling and managing suspected cases.

After the confirmation of the first COVID-19 case, SLFETP immediately deployed 12 graduates at three main points of entry, including Lungi International Airport, Guinea, and Liberia land borders. They supported the MoH in implementing and managing the quarantine process for travelers from countries reporting 50 or more cases of COVID-19. Because of the critical support of FETP to the MoH in implementing and managing quarantined travelers, the first case of COVID-19 was identified by an FETP graduate, a Sierra Leonean who traveled to France on March 12, 2020 and returned to Sierra Leone on March 16, 2020, through Lungi International Airport. By the time the first case was detected, 569 travelers had been quarantined and monitored with the support of FETP graduates. The deployed FETP graduates analyzed quarantine data daily and shared it with the national surveillance program for dissemination at the national COVID-19 preparedness and response coordination meetings (17).

In the response phase of COVID-19, the quarantine protocol was reviewed six times with SLFETP graduates and technical staff support, considering the four WHO COVID-19 transmission scenarios (18). Also, SLFETP graduates held key roles such as data management within the quarantine sub-pillar at both district and national levels. They continued implementing quarantine activities, analyzing and disseminating relevant data about quarantined people, and building the capacity of other MoH quarantine staff. SLFETP graduates trained 170 quarantine officers on the quarantine protocol. Cumulatively, 7,349 contacts and travelers were quarantined, and 5,748 were discharged following the quarantine protocol.

SLFETP graduates created key documents to enhance COVID-19 quarantine management in Sierra Leone. These included an information sheet for those in quarantine, an assessment tool for facilities, a register for managing locations, and a daily monitoring checklist for facility managers. They also developed reporting forms for quarantine officers and standard operating procedures (SOPs) for home/self-quarantine and the quarantine of healthcare workers.

After the first COVID-19 case was declared in the country on March 31^st^, 2020, 11 FETP trainees were deployed to the Lungi International Airport, and the number of screening points increased from 3 to 24 ground crossing points, seaports, and wharves. Approximately 120 points of entry staff trained by the International Center for AIDS Care and Treatment Programs (ICAP) in collaboration with FETP were deployed and supervised by 14 FETP graduates. As of June 2022, more than 10,000 passengers had been screened at points of entry. The national point of entry coordinator commented that “*heightening surveillance activities at PoE had surely helped us in addressing some of those challenges relating to effective surveillance, thereby improving prevention, detection, reporting and immediate response to diseases of Public Health importance emerging from the border points*.”

#### Enhanced surveillance

A total of 24 FETP-Intermediate graduates were mobilized to provide surge capacity for enhanced surveillance of COVID-19. Enhanced COVID-19 surveillance involved testing all suspected respiratory illness cases in selected healthcare facilities, offering COVID-19 tests for every client visiting selected sentinel healthcare facilities, and testing randomly chosen people in hotspot communities.

### Investigating COVID-19 cases

Graduates and trainees of the FETP-Frontline and FETP-Intermediate analyzed epidemiological data to ensure that interventions were data-driven. Early detection, and timely investigation, isolation of cases, alerts, and contact identification were the core activities of the FETP trainees and graduates who were the frontline responders of the pandemic. SLFETP-Intermediate and Frontline graduates and trainees constructed the first COVID-19 transmission chain (17). As of June 2022, they traced the source of infection for 56% of the 7,762 cases, and their contacts were identified and placed in quarantine or isolation (17).

The national COVID-19 case investigation coordinator at the MOH and a graduate of the FETP said, “*Having this FETP course for us, Sierra Leoneans, is critical for such outbreaks because we want to have a pool of competent frontline epidemiologists and experts in outbreak investigation and response on whom the country can rely to tackle this type of public health emergency and others and to build a resilient and responsive public health system*.” The coordinator added that during a supervisory visit to district level FETP responders that “*if we had been trained in FETP during the Ebola outbreak, we would have controlled it early and we would not have lost thousands of Sierra Leonean lives*.”

### Strengthening contact tracing of COVID-19 cases

Contact tracing was conducted by FETP trainees and graduates and Community Health Workers. Contact tracing aimed to rapidly identify potential secondary cases infected by known primary cases and institute containment measures to avoid continuous community spread in affected communities (19). The SLFETP trainees and graduates, with the technical guidance of the FETP faculty, developed contact tracing tools, prepared training materials on COVID-19, and trained FETP graduates and trainees on contact tracing. In the early phase of the outbreak, SLFETP trainees and graduates trained 410 contact tracers in Western Area Urban and 200 in Western Area rural districts. They were deployed to the field to monitor contacts under the supervision of FETP graduates and trainees. As the outbreak evolved and spread to other districts, the SLFETP faculty, in collaboration with other partners, conducted a training of trainers for 42 FETP graduates and trainees from 14 districts (3 per district). They then cascaded the training of contact tracers in their respective districts. A total of 100 community health workers and ten peer supervisors per district were also trained. SLFETP graduates and trainees coordinated and supervised contact tracing activities at national and district levels under the technical support of SLFETP mentors. Since 85% of all confirmed cases were asymptomatic, many of the cases were detected through contact tracing; that is, by testing asymptomatic individuals who were contacts of COVID-19 confirmed cases. As of August 4, 2022, 18,686 contacts had been identified, line-listed, and monitored; cumulatively, 378,511 laboratory tests were performed (12).

### Epidemiological and operational research for COVID-19

The SLFETP conducted several operational research surveys during the COVID-19 response, including the first few cases study to investigate the epidemiological characteristics of COVID-19 in the early phase of the response (20), a survey on the knowledge, attitude, and practices on protective behavioral practices against COVID-19 among health workers (21), and behavioral practices toward antibiotic use among healthcare workers (22). The FETP graduates led the first few cases study. The study investigated the clinical and epidemiological characteristics of COVID-19 in the early phase of the response, which aimed to understand the key clinical characteristics of COVID-19 in the country, including its clinical severity and proportion of symptomatic cases, and to understand the key epidemiological features of the COVID-19 disease. This study's findings helped the Sierra Leone government revise its response policies and strategies to reduce COVID-19-related deaths.

### Response to outbreaks of infectious diseases other than COVID during the COVID-19 pandemic

During the COVID-19 pandemic, the graduates and trainees of Intermediate and Frontline FETP levels responded to five non-COVID-19 outbreaks: measles, monkeypox, rabies, Lassa fever, and polio. The FETP-Intermediate and Frontline trainees and graduates investigated alerts, cases, and events of national and international concern, such as the suspected Marburg virus, maternal deaths, and acute flaccid paralysis (23, 24).

#### FETP's role in addressing other challenges

The SLFETP played a critical role at the start of the COVID-19 pandemic by addressing key challenges, such as the lack of training materials for surveillance and healthcare workers and community health workers and the need for effective training to respond to the pandemic. Additional issues included stigma, denial, and non-adherence to preventive measures. To overcome these challenges, the SLFETP faculty created training materials focused on identifying suspected cases, screening travelers, conducting case investigations, contact tracing, and enhancing social mobilization and risk communication.

## Discussion

The Sierra Leone FETP trainees, graduates, and staff contributed vitally to the preparedness and response to the COVID-19 pandemic. The FETP program equips trainees with core competencies that enable them to actively engage and lead outbreak investigations, manage public health surveillance and data, communicate risk effectively, and conduct surveys that help define populations at risk and interventions that are most impactful. Many of these competencies were both needed and used during the response to the pandemic.

In many African countries, including Uganda, Ethiopia, Kenya, Ghana, Nigeria, and Guinea, FETP graduates and trainees were involved in several COVID-19 preparedness and response-related activities such as enhancing surveillance for early detection and notification, case investigation and contact tracing, screening of passengers at airports and land crossings; data collection, management, and analysis; and risk communications (8, 9, 11, 25, 26). In Jordan, Yemen, Egypt, and Sudan, FETP graduates played crucial roles in managing the COVID-19 pandemic (8, 27). The Central American FETP was actively involved in the COVID-19 preparedness and response activities, such as developing a protocol for detection, evaluation, and response to potential COVID-19 cases. FETP graduates played critical roles in preparing daily reports for decision-makers, developing recommendations for health teams, travelers, and points of entry, and conducting surveillance. The graduates also played key roles in data analysis and information communication for action (17). Many countries utilize FETP graduates to strengthen their public health workforce and respond to emergencies (9, 26).

The International Health Regulations (IHR) 2005 highlight the need for countries to develop capacities to prevent, detect, and respond to outbreaks. Local capacity is crucial for timely detection and effective response to public health emergencies or events of national or international concern. Currently, in Sierra Leone, each district has at least three district surveillance officers trained through FETP. These officers are responsible for managing surveillance and are the frontline responders to any public health emergency.

SLFETP has increased the trained workforce at the district level. All 16 districts in the country have at least one surveillance officer trained through the Intermediate level of the SLFETP, compared to none at the program's inception in 2016. The roles played by SLFETP trainees and graduates during the preparedness and response phases of the COVID-19 pandemic are clear evidence of the country's capacity to fulfill the IHR core capacities, particularly the capacity to detect, verify, and notify public health emergencies at designated points of entry.

In 2016, Sierra Leone had limited capacity to respond to the EVD outbreak, and almost all districts were supported by international experts. The IHR monitoring and evaluation framework and the Joint External Evaluation (JEE) call for countries to have at least two of the three levels of FETP to demonstrate capacities in the human resource technical area of Global Health Security. Sierra Leone has two FETP tiers, frontline and intermediate levels, thus fulfilling the IHR and JEE requirements. The advanced level of FETP, through the Ghana West Africa regional FETP, is available to Sierra Leoneans. Sierra Leone has 13 field epidemiologists who graduated from the Ghana Regional Advanced Field Epidemiology and Laboratory Training Program.

The active engagement of FETP trainees, graduates, and staff in the preparedness and response actions to outbreaks such as the COVID-19 pandemic demonstrated the importance of building public health workforce capacity, particularly in low- and middle-income countries (28). Building the capacity of public health professionals through the field epidemiology program has a vital role in responding to public health emergencies like the COVID-19 pandemic. In Sierra Leone, the FETP trainees and graduates successfully implemented activities related to COVID-19 preparedness and response (29).

The limitation of this study is that the review of the documents and interviews to document the role of the FETP started in August 2022, 2 years after the pandemic began. This might have contributed to potential recall bias in the interviews. Therefore, the actual roles and contributions of the Sierra Leone FETP during the preparedness and response phases of the pandemic might have been underrepresented due to recall bias and lack of or misplaced documentation. In addition, the dates of the activities were not systematically recorded and made it challenging to accurately describe the events chronologically.

## Conclusions

The Sierra Leone FETP has been critical to the MOH during the preparedness and response phase of the COVID-19 pandemic. The trainees and graduates have enhanced the surveillance system and led key pillars of the COVID-19 response, mainly coordination, surveillance, laboratory, risk communication, and quarantine. FETP supported various districts by building their capacity, especially in the district surveillance pillar, to conduct case investigation, contact tracing and quarantine monitoring, and data management.

The public health capacity built through the FETP is a critical asset to the country. These efforts and gains should be maintained, sustained, and scaled -up by supporting the program to build and maintain a strong and resilient public health workforce in Sierra Leone, which is crucial for successfully managing future outbreaks. We recommend the SLFETP develop tools and document the day-to-day activities, achievements, and contributions of FETP during future outbreaks or pandemics.

## Data Availability

The original contributions presented in the study are included in the article/supplementary material, further inquiries can be directed to the corresponding authors.
